# Supplementary material for: A Comprehensive Molecular Characterization of the Pancreatic Neuroendocrine Tumor Cell Lines BON-1 and QGP-1
Source: Cancers (Basel). 2020 Mar 14;12(3):691. doi: 10.3390/cancers12030691 (PMC7140066; doi:10.3390/cancers12030691)

*Supplementary material*

A comprehensive molecular characterization of the pancreatic neuroendocrine tumor cell lines BON-1 and QGP-1

Kim B. Luley, Shauni B. Biedermann, Axel Künstner, Hauke Busch, Sören Franzenburg, Jörg Schrader, Patricia Grabowski, Ulrich F. Wellner, Tobias Keck, Georg Brabant, Sebastian M. Schmid, Hendrik Lehnert, and Hendrik Ungefroren

| **MiR** | **Biological function** | **Reference** |
| --- | --- | --- |
| 1251 | -potential prognostic marker in head and neck squamous cell carcinoma, targets IGF1 | Hui L et al. 2016 |
| 7-3 | -highly expressed in both the developing and adult pancreas  -potential role in endocrine cell differentiation and function  -upregulates insulin transcription, expression, and secretion in cultured β-cells and isolated primary islets  -negatively regulates the mTOR pathway and proliferation in adult pancreatic β-cells | Correa-Medina et al. 2009, Joglekar et al. 2009, Ebrahimi et al. 2016, Wang Y et al. 2013 |
| 1228 | -stably expressed reference miR in exosomes, plasma and tissue from colorectal cancer patients and healthy controls | Danese et al. 2017 |
| 15a | -critical for proliferation of pancreatic β-cells  -positively regulates insulin synthesis  -binds to Ngn3 mRNA during pancreas regeneration  -dysregulated in PanIN lesions  -inhibits cell proliferation and EMT in PDAC | Sun et al. 2011, Yu J et al. 2012, Joglekar et al. 2007, Guo et al. 2014 |
| 182 | -potential role in endocrine cell differentiation and/or function  -upregulates INS transcription, expression, and secretion in isolated primary islets  -dysregulated in PanIN lesions  -promotes pancreatic cancer cell proliferation and migration  -potential diagnostic and prognostic marker for PDAC | Yu J et al. 2012, Joglekar et al. 2009, Melkmann-Zehavi et al. 2011, Chen et al. 2014, Wang et al. 2106 |
| 3609 | -functions as a chemosensitizer by blocking the PDL-1 immune checkpoint  -differentially expressed in PDAC between paired tumor and adjacent benign tissue  -upregulated after GDNF treatment of glioma cells | Li D et al. 2019, Mao et al. 2017, Zhang BL et al. 2017 |
| 3653 | -inhibits metastasis and EMT in colon cancer  -negatively associated with larger tumor size, lymph node metastasis and poor survival in glioma  -upregulated in panNET with distant metastases compared to those with locoregional disease  -associated with *ATRX* abnormalities  -potential tissue biomarker for increased metastatic risk in panNET | Zhu et al. 2019, Chen, Li et al. 2019, Gill et al. 2019 |
| 664b | -suppresses tumor growth and metastasis of PDAC cells  -suppresses cell proliferation and invasion in breast cancer  -low expression associated with poor OS and DFS  -increased in serum samples of patients with primary liver cancer  -dysregulated in PanIN lesions | Gill et al. 2019, Li W et al. 2018, Wu et al. 2019, Yu et al. 2012 |
| 221 | -upregulated in several types of human tumors  -acts as oncogene or tumor suppressor  -therapeutic modulator in PDAC | Chakraborty et al. 2013, Ravegnini et al. 2019, Ebrahimi et al. 2016 |
| 181D | -overexpressed in PDAC, downregulation suppresses PDAC development | Zhang G et al. 2017 |
| (miR-17-92-cluster)  17,18A,19A,19B1, 20A,92 | -miR-17 promotes pancreatic β-cell proliferation  -miR-18a regulates pancreatic β-cell proliferation and adaptation, expression of Ptf1a in pancreatic progenitors, and counteracts AKT and ERK activation to inhibit the proliferation of pancreatic progenitors  -regulates multiple aspects of pancreatic tumor development and progression  -miR-19a-3p enhances pancreatic β-cell proliferation and insulin secretion, and inhibits apoptosis  -miR-19b downregulates *INS1* through targeting NeuroD and is dysregulated in PanIN lesions  -miR-20a overexpression inhibits proliferation and metastasis of PDAC cells and acts as an oncogene in association with *MYC*  -miR-92a regulates insulin biosynthesis in rats | Mandelbaum et al. 2019, Chen Y et al. 2016, Lu et al. 2015, Zhang ZW et al. 2011, Setyowati, Karolina et al. 2013, Li X et al. 2017, Xiang et al. 2010, Quattrochi et al. 2017, He et al. 2005, Li X et al. 2017, Ebrahimi et al. 2016, Yu et al. 2012 |
| 21 | -enhances insulin sensitivity via the PI3K/AKT pathway  -associated with tumor proliferation, advanced stage, metastases, tumor recurrence, and reduced OS  -represses PTEN expression  -dysregulated in PanIN lesions  -potential biomarker for GEP-NET  -elevated levels in pancreatic cyst fluid are predictive of mucinous precursor lesions of PDAC | Zimmermann et al. 20018, Zhang BH et al. 2019, Malczewska A et al. 2018, Ebrahimi et al. 2016, Ryu et al. 2011, Yu et al. 2012 |
| 33A | -regulates insulin secretion and sensitivity  -modulates expression of IRS-2 and AKT phosphorylation | Zhang BH et al. 2019 |
| 1248 | -prognostic marker in breast cancer | Tanic et al. 2015 |
| 129-1 | -acts as a tumor suppressor and induces cell cycle arrest in glioblastoma multiforme  -associated with OS in PDAC | Kouhkan et al. 2016, Zhang Z et al. 2018 |
| 129-2 | -downregulated in pancreatic cancer | Ali et al. 2015 |
| 146a | -reduced levels associated with insulin resistance and poor glycemic control in T2D  -promotes proliferation, migration and invasion in lung cancer cells  -suppresses NFκB activity with reduction of metastatic potential in breast cancer cells | Bhaumik et al. 2008, Kaviani et al. 2016, Chakraborty et al. 2013, Balasubramanyam et al. 2011, Yu et al. 2012 |
| 375 | -controls growth and differentiation of islet cells, formation of β-cell identity and mass, production and secretion of insulin  -targets Gata6, Hnf1β, Pax6 and is itself targeted by Ngn3, NeuroD1, Pdx1 and Hnf6 | Eliasson et al. 2017, Zhang BH et al. 2019, Joglekar et al. 2009 |
| 4449 | -downregulated in peripheral blood of lung cancer patients  -upregulated in peripheral blood of multiple myeloma patients | He et al. 2010, Shen et al. 2017 |
| 3131 | -3-bp indel polymorphism associated with increased risk of breast cancer | Azizi et al. 2019 |
| let-7a-3, let‑7f‑1 | -inhibits proliferation and induces apoptosis by targeting AKT2 in prostate cancer cells  -affects chemosensitivity in PDAC  -reduces the vasculogenic mimicry of human glioma cells  -overexpression associated with better OS in PDAC and poor OS in non-small cell lung cancer | Lamichhane et al. 2018, Bhutia et al. 2013, Xue et al. 2016, Ali et al. 2015 |
| let-7b | -suppresses human pancreatic cancer stem cell proliferation and invasion | Shao et al. 2015 |
| 877 | -potential diagnostic and prognostic biomarker for PDAC | Su et al. 2018 |

**Table S2.** List of miRs identified in BON-1, QGP-1 and NT-3 cells and their functional activities. Only miRs with relative expression values > 2 have been included here. OS, overall survival; PFS, progression-free survival. See main manuscript text for details.

**References**

Ali, S.; Dubaybo, H.; Brand, R.E.; Sarkar, F.H. Differential Expression of MicroRNAs in Tissues and Plasma Co-exists as a Biomarker for Pancreatic Cancer. *J. Cancer Sci. Ther*. **2015**, *7*, 336-346.

Azizi, M.; Rahimi, N.; Bahari, G.; Hashemi, S.M.; Hashemi, M. The relationship between pre-miR-3131 3-bp insertion/deletion polymorphism and susceptibility and clinicopathological characteristics of patients with breast cancer. *Microrna* **2019** Sep 6.

Balasubramanyam, M.; Aravind, S.; Gokulakrishnan, K.; Prabu, P.; Sathishkumar, C.; Ranjani, H.; Mohan, V. Impaired miR-146a expression links subclinical inflammation and insulin resistance in Type 2 diabetes. *Mol. Cell. Biochem*. **2011**, *351*, 197-205.

Bhaumik, D.; Scott, G.K.; Schokrpur, S.; Patil, C.K.; Campisi, J.; Benz, C.C. Expression of microRNA-146 suppresses NF-kappaB activity with reduction of metastatic potential in breast cancer cells. *Oncogene* **2008**, *27*, 5643-5647.

Bhutia, Y.D.; Hung, S.W.; Krentz, M.; Patel, D.; Lovin, D.; Manoharan, R.; Thomson, J.M.; Govindarajan, R. Differential processing of let-7a precursors influences RRM2 expression and chemosensitivity in pancreatic cancer: role of LIN-28 and SET oncoprotein. *PLoS One* **2013**, *8*, e53436.

Chakraborty, C.; George Priya Doss C.; Bandyopadhyay, S. miRNAs in insulin resistance and diabetes-associated pancreatic cancer: the 'minute and miracle' molecule moving as a monitor in the 'genomic galaxy'. *Curr. Drug Targets* **2013**, *14*, 1110-1117.

Chen, Q.; Yang, L.; Xiao, Y.; Zhu, J.; Li, Z. Circulating microRNA-182 in plasma and its potential diagnostic and prognostic value for pancreatic cancer. *Med. Oncol*. **2014**, *31*, 225.

[Chen, Y](https://www.ncbi.nlm.nih.gov/pubmed/?term=Chen%20Y%5BAuthor%5D&cauthor=true&cauthor_uid=31378901).; Li, Z.H.; Liu, X.; Liu, G.X.; Yang, H.M.; [Wu, P.F](https://www.ncbi.nlm.nih.gov/pubmed/?term=Wu%20PF%5BAuthor%5D&cauthor=true&cauthor_uid=31378901). Reduced expression of miR-3653 in glioma and its correlations with clinical progression and patient survival. *Eur. Rev. Med. Pharmacol. Sci*. **2019**, *23*, 6596-6601.

Chen, Y.; Tian, L.; Wan, S.; Xie, Y.; Chen, X.; Ji, X.; Zhao, Q.; Wang, C.; Zhang, K.; Hock, J.M.; et al. MicroRNA-17-92 cluster regulates pancreatic beta-cell proliferation and adaptation. *Mol. Cell. Endocrinol*. **2016**, *437*, 213-223.

Correa-Medina, M.; Bravo-Egana, V.; Rosero, S.; Ricordi, C.; Edlund, H.; Diez, J.; Pastori, R.L. MicroRNA miR-7 is preferentially expressed in endocrine cells of the developing and adult human pancreas. *Gene Expr. Patterns* **2009**, *9*, 193-199.

Danese, E.; Minicozzi, A.M.; Benati, M.; Paviati, E.; Lima-Oliveira, G.; Gusella, M.; Pasini, F.; Salvagno, G.L.; Montagnana, M.; Lippi, G. Reference miRNAs for colorectal cancer: analysis and verification of current data. *Sci. Rep*. **2017**, *7*, 8413.

Ebrahimi, S.; Hosseini, M.; Ghasemi, F.; Shahidsales, S.; Maftouh, M.; Akbarzade, H.; Parizadeh, S.A.; Hassanian, S.M.; Avan, A. Circulating microRNAs as Potential Diagnostic, Prognostic and Therapeutic Targets in Pancreatic Cancer. *Curr. Pharm. Des*. **2016**, *22*, 6444-6450.

Gill, P.; Kim, E.; Chua, T.C.; Clifton-Bligh, R.J.; Nahm, C.B.; Mittal, A.; Gill, A.J.; Samra, J.S. MiRNA-3653 Is a Potential Tissue Biomarker for Increased Metastatic Risk in Pancreatic Neuroendocrine Tumours. *Endocr. Pathol*. **2019**, *30*, 128-133.

Guo, S.; Xu, X.; Tang, Y.; Zhang, C.; Li, J.; Ouyang, Y.; Ju, J.; Bie, P.; Wang, H. miR-15a inhibits cell proliferation and epithelial to mesenchymal transition in pancreatic ductal adenocarcinoma by down-regulating Bmi-1 expression. *Cancer Lett*. **2014**, *344*, 40-46.

He, L.; Thomson, J.M.; Hemann, M.T.; Hernando-Monge, E.; Mu, D.; Goodson, S.; Powers, S.; Cordon-Cardo, C.; Lowe, S.W.; Hannon, G.J.; et al. A microRNA polycistron as a potential human oncogene. *Nature* **2005**, *435*, 828-833.

He, Q.; Fang, Y.; Lu, F.; Pan, J.; Wang, L.; Gong, W.; Fei, F.; Cui, J.; Zhong, J.; Hu, R.; et al. Analysis of differential expression profile of miRNA in peripheral blood of patients with lung cancer. *J. Clin. Lab. Anal*. **2019**, *33*, e23003.

Hui, L.; Wu, H.; Yang, N.; Guo, X.; Jang, X. Identification of prognostic microRNA candidates for head and neck squamous cell carcinoma. *Oncol. Rep*. **2016**, *35*, 3321-3330.

Joglekar, M.V.; Joglekar, V.M.; Hardikar, A.A. Expression of islet-specific microRNAs during human pancreatic development. *Gene Expr. Patterns* **2009**, *9*, 109-113.

Joglekar, M.V.; Parekh, V.S.; Mehta, S.; Bhonde, R.R.; Hardikar, A.A. MicroRNA profiling of developing and regenerating pancreas reveal post-transcriptional regulation of neurogenin3. *Dev. Biol*. **2007**, *311*, 603-612.

Kaviani, M.; Azarpira, N.; Karimi, M.H.; Al-Abdullah, I. The role of microRNAs in islet β-cell development. *Cell Biol. Int*. **2016**, *40*, 1248-1255.

Kouhkan, F, Mobarra, N.; Soufi-Zomorrod, M.; Keramati, F.; Hosseini, Rad S.M.; Fathi-Roudsari, M.; Tavakoli, R.; Hajarizadeh, A.; Ziaei, S.; Lahmi, R.; et al. MicroRNA-129-1 acts as tumour suppressor and induces cell cycle arrest of GBM cancer cells through targeting IGF2BP3 and MAPK1. *J. Med. Genet*. **2016**, *53*, 24-33.

Lamichhane, S.R.; Thachil, T.; De Ieso, P.; Gee, H.; Moss, S.A.; Milic, N. Prognostic Role of MicroRNAs in Human Non-Small-Cell Lung Cancer: A Systematic Review and Meta-Analysis. *Dis. Markers* **2018**, *2018*, 8309015.

Li, D.; Wang, X.; Yang, M.; Kan, Q.; Duan, Z. miR3609 sensitizes breast cancer cells to adriamycin by blocking the programmed death-ligand 1 immune checkpoint. *Exp. Cell Res*. **2019**, *380*, 20-28.

Li, W.; Yu, Z.X.; Ma, B.F. The increase of miR-27a affects the role of cisplatin on proliferation and migration capacities of liver cancer cells. *Eur. Rev. Med. Pharmacol. Sci*. **2018**, *22*, 5490-5498.

Li, X.; Zhang, Z.; Li, Y.; Zhao, Y.; Zhai, W.; Yang, L.; Kong, D.; Wu, C.; Chen, Z.; Teng, C.B. miR-18a counteracts AKT and ERK activation to inhibit the proliferation of pancreatic progenitor cells. *Sci. Rep*. **2017**, *7*, 45002.

Lu, Y.; Fei, X.Q.; Yang, S.F.; Xu, B.K.; Li, Y.Y. Glucose-induced microRNA-17 promotes pancreatic beta cell proliferation through down-regulation of Menin. *Eur. Rev. Med. Pharmacol. Sci*. **2015**, *19*, 624-9.

Mandelbaum, A.D.; Kredo-Russo, S.; Aronowitz, D.; Myers, N.; Yanowski, E.; Klochendler, A.; Swisa, A.; Dor, Y.; Hornstein, E. miR-17-92 and miR-106b-25 clusters regulate beta cell mitotic checkpoint and insulin secretion in mice. *Diabetologia* **2019**, *62*, 1653-1666.

Malczewska, A.; Kidd, M.; Matar, S.; Kos-Kudla, B.; Modlin, I.M. A Comprehensive Assessment of the Role of miRNAs as Biomarkers in Gastroenteropancreatic Neuroendocrine Tumors. *Neuroendocrinology* **2018**, *107*, 73-90.

Mao, Y.; Shen, J.; Lu, Y.; Lin, K.; Wang, H.; Li, Y.; Chang, P.; Walker, M.G.; Li, D. RNA sequencing analyses reveal novel differentially expressed genes and pathways in pancreatic cancer. *Oncotarget* **2017**, *8*, 42537-42547.

Melkman-Zehavi, T.; Oren, R.; Kredo-Russo, S.; Shapira, T.; Mandelbaum, A.D.; Rivkin, N.; Nir, T.; Lennox, K.A.; Behlke, M.A.; Dor, Y.; et al. miRNAs control insulin content in pancreatic β-cells via downregulation of transcriptional repressors. *EMBO J*. **2011**, *30*, 835-845.

Quattrochi, B.; Gulvady, A.; Driscoll, D.R.; Sano, M.; Klimstra, D.S.; Turner, C.E.; Lewis, B.C. MicroRNAs of the mir-17~92 cluster regulate multiple aspects of pancreatic tumor development and progression. *Oncotarget* **2017**, *8*, 35902-35918.

Ravegnini, G.; Cargnin, S.; Sammarini, G.; Zanotti, F.; Bermejo, J.L.; Hrelia, P.; Terrazzino, S.; Angelini, S. Prognostic Role of miR-221 and miR-222 Expression in Cancer Patients: A Systematic Review and Meta-Analysis. *Cancers (Basel)* **2019**, *11*, pii: E970.

Ryu, J.K.; Matthaei, H.; Dal Molin, M.; Hong, S.M.; Canto, M.I.; Schulick, R.D.; Wolfgang, C.; Goggins, M.G.; Hruban, R.H.; Cope, L.; et al. Elevated microRNA miR-21 levels in pancreatic cyst fluid are predictive of mucinous precursor lesions of ductal adenocarcinoma. *Pancreatology* **2011**, *11*, 343-350.

Setyowati Karolina, D.; Sepramaniam, S.; Tan, H.Z.; Armugam, A.; Jeyaseelan, K. miR-25 and miR-92a regulate insulin I biosynthesis in rats. *RNA Biol*. **2013**, *10*, 1365-1378.

Shao, Y.; Zhang, L.; Cui, L.; Lou, W.; Wang, D.; Lu, W.; Jin, D.; Liu, T. LIN28B suppresses microRNA let-7b expression to promote CD44+/LIN28B+ human pancreatic cancer stem cell proliferation and invasion. *Am. J. Cancer Res*. **2015**, *5*, 2643-2659.

Shen, X.; Ye, Y.; Qi, J.; Shi, W.; Wu, X.; Ni, H.; Cong, H.; Ju, S. Identification of a novel microRNA, miR-4449, as a potential blood based marker in multiple myeloma. *Clin. Chem. Lab. Med*. **2017**, *55*, 748-754.

Su, Q.; Zhu, E.C.; Qu, Y.L.; Wang, D.Y.; Qu, W.W.; Zhang, C.G.; Wu, T.; Gao, Z.H. Serum level of co-expressed hub miRNAs as diagnostic and prognostic biomarkers for pancreatic ductal adenocarcinoma. *J. Cancer* **2018**, *9*, 3991-3999.

Sun, L.L.; Jiang, B.G.; Li, W.T.; Zou, J.J.; Shi, Y.Q.; Liu, Z.M. MicroRNA-15a positively regulates insulin synthesis by inhibiting uncoupling protein-2 expression. *Diabetes Res. Clin. Pract*. **2011**, *91*, 94-100.

Tanic, M. ; Yanowski, K. ; Gómez-López, G. ; Rodriguez-Pinilla, M.S. ; Marquez-Rodas, I. ; Osorio, A. ; Pisano, D.G. ; Martinez-Delgado, B. ; Benítez, J. MicroRNA expression signatures for the prediction of BRCA1/2 mutation-associated hereditary breast cancer in paraffin-embedded formalin-fixed breast tumors. *Int. J. Cancer* **2015**, *136*, 593-602.

Wang, S.; Ji, J.; Song, J.; Li, X.; Han, S.; Lian, W.; Cao, C.; Zhang, X.; Li, M. MicroRNA-182 promotes pancreatic cancer cell proliferation and migration by targeting β-TrCP2. *Acta Biochim. Biophys. Sin. (Shanghai)* **2016**, *48*, 1085-1093.

Wang, Y.; Liu, J.; Liu, C.; Naji, A.; Stoffers, D.A. MicroRNA-7 regulates the mTOR pathway and proliferation in adult pancreatic β-cells. *Diabetes* **2013**, *62*, 887-895.

Wu, L.; Li, Y.; Li, J.; Ma, D. MicroRNA-664 Targets Insulin Receptor Substrate 1 to Suppress Cell Proliferation and Invasion in Breast Cancer. *Oncol. Res*. **2019**, *27*, 459-467.

Xiang, J.; Wu, J. Feud or Friend? The Role of the miR-17-92 Cluster in Tumorigenesis. *Curr. Genomics* **2010**, *11*, 129-135.

Xue, H.; Gao, X.; Xu, S.; Zhang, J.; Guo, X.; Yan, S.; Li, T.; Guo, X.; Liu, Q.; Li, G. MicroRNA-Let-7f reduces the vasculogenic mimicry of human glioma cells by regulating periostin-dependent migration. *Oncol. Rep*. **2016**, *35*, 1771-1777.

Yu, J.; Li, A.; Hong, S.M.; Hruban, R.H.; Goggins, M. MicroRNA alterations of pancreatic intraepithelial neoplasias. *Clin. Cancer Res*. **2012**, *18*, 981-992.

Zhang, B.L.; Dong, F.L.; Guo, T.W.; Gu, X.H.; Huang, L.Y.; Gao, D.S. MiRNAs Mediate GDNF-Induced Proliferation and Migration of Glioma Cells. *Cell. Physiol. Biochem*. **2017**, *44*, 1923-1938.

Zhang, B.H.; Shen, C.A.; Zhu, B.W.; An, H.Y.; Zheng, B.; Xu, S.B.; Sun, J.C.; Sun, P.C.; Zhang, W.; Wang, J.; et al. Insight into miRNAs related with glucometabolic disorder. *Biomed. Pharmacother*. **2019**, *111*, 657-665.

Zhang, G.; Liu, D.; Long, G.; Shi, L.; Qiu, H.; Hu, G.; Hu, G.; Liu, S. Downregulation of microRNA-181d had suppressive effect on pancreatic cancer development through inverse regulation of KNAIN2. *Tumour Biol*. **2017**, *39*, 1010428317698364.

Zhang, Z.; Pan, B.; Lv, S.; Ji, Z.; Wu, Q.; Lang, R.; He, Q.; Zhao, X. Integrating MicroRNA Expression Profiling Studies to Systematically Evaluate the Diagnostic Value of MicroRNAs in Pancreatic Cancer and Validate Their Prognostic Significance with the Cancer Genome Atlas Data. *Cell. Physiol. Biochem*. **2018**, *49*, 678-695.

Zhang, Z.W.; Zhang, L.Q.; Ding, L.; Wang, F.; Sun, Y.J.; An, Y.; Zhao, Y.; Li, Y.H.; Teng, C.B. MicroRNA-19b downregulates insulin 1 through targeting transcription factor NeuroD1. *FEBS Lett*. **2011**, *585*, 2592-2598.

Zhu, W.; Luo, X.; Fu, H.; Liu, L.; Sun, P.; Wang, Z. MiR-3653 inhibits the metastasis and epithelial-mesenchymal transition of colon cancer by targeting Zeb2. *Pathol. Res. Pract*. **2019**, *215*, 152577.

Zimmermann, N.; Knief, J.; Kacprowski, T.; Lazar-Karsten, P.; Keck, T.; Billmann, F.; Schmid, S.; Luley, K.; Lehnert, H.; Brabant, G.; et al. MicroRNA analysis of gastroenteropancreatic neuroendocrine tumors and metastases. *Oncotarget* **2018**, *9*, 28379-29390.

| **Primer name** | **Sequence (5’→3’)** | **GenBank accession** |
| --- | --- | --- |
| ALK7-forward | caacaacataacactgcaccttcc | NM_145259 |
| ALK7-reverse | tttcatgtcgcagcatgaccgtc | NM_145259 |
| CDX2-forward | aagtgtcccagagcccttgag | NM_001265 |
| CDX2-reverse | ccacttgtcttactcctggctc | NM_001265 |
| CgA-forward | taaaggggataccgaggtgatg | J03483.1 |
| CgA-reverse | tcggagtgtctcaaaacattcc | J03483.1 |
| Insulin-forward | gcagcctttgtgaaccaacac | NM_000207 |
| Insulin-reverse | ccccgcacactaggtagaga | NM_000207 |
| Glucagon-forward | acagagcttaggacacagagc | NM_002054 |
| Glucagon-reverse | caggtgatgttgtgaagatgatc | NM_002054 |
| GATA4-forward | tggcatctggtatcggagtga | NM_002052 |
| GATA4-reverse | gctggaaatctgatgggcac | NM_002052 |
| GATA6-forward | gtgccaactgtcacaccaca | NM_005257 |
| GATA6-reverse | gagtccacaagcattgcacac | NM_005257 |
| NGN3-forward | ctccagggtagaaaggatgacgcctc | NM_020999 |
| NGN3-reverse | acccgtgaatgggattatggggtggtg | NM_020999 |
| IPF1/PDX1-forward | cccatggatgaagtctacc | NM_000209 |
| IPF1/PDX1-reverse | gtcctcctcctttttccac | NM_000209 |
| NKX2.2-forward | aggaggcctcggtccttatgg | NM_002509 |
| NKX2.2-reverse | agcgaagctgcgcaaacattctg | NM_002509 |
| PAX6-forward | gaatcagagaagacaggccag | NM_000280 |
| PAX6-reverse | gggtgtaggtatcataactccg | NM_000280 |
| PAX4-forward | gaggacacggtgagggtctgg | AF043978 |
| PAX4-reverse | aggggacagtgggaggaaggg | AF043978 |
| FOXA2/HNF3β-forward | caccaaccccacaaaatggacc | AB028021 |
| FOXA2/HNF3β-reverse | ctctctcacttgtcctcgatcc | AB028021 |
| ISL1-forward | gttgtacgggatcaaatgcgc | NM_002202 |
| ISL1-reverse | gtcattgggctgctgctgc | NM_002202 |
| MafA-forward | cgagctgaaccggcagctc | NM_201589 |
| MafA-reverse | gccagcttctcgtatttctccttg | NM_201589 |
| MAP2-forward | catcatacgtactcctccaaaatctc | U01828.1 |
| MAP2-reverse | attcacaagccctgcttagcgag | U01828.1 |
| NCAM1-forward | aggagacagaaacgaagcca | NM_000615 |
| NCAM1-reverse | ggtgttggaaatgctctggt | NM_000615 |
| NEFL-forward | ccaagacctcctcaacgtgaag | NM_006158 |
| NEFL-reverse | ccagcaccttcaactttcttctcc | NM_006158 |
| NeuroD1-forward | accatgaccaaatcgtacagcgag | NM_002500 |
| NeuroD1-reverse | tggaagacatgggagctgtc | NM_002500 |
| NKX6.1-forward | ccggacagcagatcttcgcc | NM_006168 |
| NKX6.1-reverse | gacttgtgcttcttcaacagctgc | NM_006168 |
| PTF1A-forward | caggacactctctctcatgga | NM_178161 |
| PTF1A-reverse | tggtggttcgttttctatgttgt | NM_178161 |
| SST-forward | ctccagctcggctttcgc | NM_001048 |
| SST-reverse | gtctcgctgaagacttggag | NM_001048 |
| SSTR2-forward | tggctatccattccatttgacc | NM_001050 |
| SSTR2-reverse | aggactgcattgcttgtcagg | NM_001050 |
| SSTR5-forward | tgtttgcgggatgttggct | NM_001053 |
| SSTR5-reverse | ctgttggcgtaggagagga | NM_001053 |
| TBP-foward | gctggcccatagtgatct tt | NM_003194 |
| TBP-reverse | cttcacacgccaagaaacag | NM_003194 |

**Table S3.** Primers used for qPCR.

© 2019 by the authors. Licensee MDPI, Basel, Switzerland. This article is an open access article distributed under the terms and conditions of the Creative Commons Attribution (CC BY) license (http://creativecommons.org/licenses/by/4.0/).
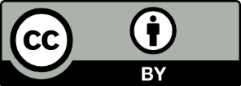

Supplement: Supplementary file 1 [file cancers-12-00691-s001.zip › cancers-730186-suppl-revised/cancers-730186-suppl-revised.docx]
